# Blood–brain barrier disruption as a cause of various serum neuron-specific enolase cut-off values for neurological prognosis in cardiac arrest patients

**DOI:** 10.1038/s41598-022-06233-4

**Published:** 2022-02-09

**Authors:** Changshin Kang, Yeonho You, Hong Joon Ahn, Jung Soo Park, Wonjoon Jeong, Jin Hong Min, Yong Nam In, Insool Yoo, Yongchul Cho, Seung Ryu, Jinwoong Lee, Seung Whan Kim

**Affiliations:** 1 Department of Emergency Medicine, Chungnam National University Hospital, 282 Munhwa-ro, Jung-gu, Daejeon, 35015 Republic of Korea; 2 Department of Emergency Medicine, College of Medicine, Chungnam National University School of Medicine, Daejeon, Republic of Korea; 3 Department of Emergency Medicine, Chungnam National University Sejong Hospital, Sejong, Republic of Korea

**Keywords:** Neuroscience, Cardiology

## Abstract

We compared the cut-off and prognostic value of serum neuron-specific enolase (NSE) between groups with and without severe blood–brain barrier (BBB) disruption to reveal that a cause of various serum NSE cut-off value for neurological prognosis is severe BBB disruption in out-of-hospital cardiac arrest (OHCA) patients underwent target temperature management (TTM). This was a prospective, single-centre study conducted from January 2019 to June 2021. Severe BBB disruption was indicated using cerebrospinal fluid-serum albumin quotient values > 0.02. The area under the receiver operating characteristic curve of serum NSE obtained on day 3 of hospitalisation to predict poor outcomes was used. In patients with poor neurologic outcomes, serum NSE in those with severe BBB disruption was higher than in those without (*P* = 0.006). A serum NSE cut-off value of 40.4 μg/L for poor outcomes in patients without severe BBB disruption had a sensitivity of 41.7% and a specificity of 96.0%, whereas a cut-off value of 34.6 μg/L in those with severe BBB disruption had a sensitivity of 86.4% and a specificity of 100.0%. We demonstrated that the cut-off and prognostic value of serum NSE were heterogeneous, depending on severe BBB disruption in OHCA patients treated with TTM.

## Introduction

Ischaemia–reperfusion cerebral injury after cardiac arrest (CA) is significantly associated with mortality and poor neurologic prognosis in CA patients^1,2^. Despite advances in cardiopulmonary resuscitation (CPR) and post-resuscitation care including target temperature management (TTM)^3^, a significant number of out-of-hospital cardiac arrest (OHCA) patients remain unconscious after treatment^4^. Because the clinical outcomes of these patients are associated with long-term hospitalization and financial burdens, early and accurate prognosis for these patients is important for selecting the most appropriate diagnostic or treatment strategy, considering withdrawal of life-sustaining therapies in hopeless patients^5,6^.

Recent guidelines recommend a multimodal strategy for prognosis in these patients. The absence of brainstem reflexes and bilateral absence of cortical somatosensory evoked potentials are the strongest predictors of poor neurologic outcomes and must be investigated first. The second line modality for predicting neurologic outcomes includes electroencephalography and imaging techniques. In addition, biomarkers of brain injury, particularly neuron-specific enolase (NSE) levels, are useful tools for predicting neurologic outcomes and considering therapeutic strategies. The European Resuscitation Council and European Society of Intensive Care Medicine Guidelines 2021 recommend a specific NSE cut-off value of 60 μg/L at 48 and/or 72 h after the return of spontaneous circulation (ROSC) to accurately predict outcomes after CA. However, repeat NSE measurements are currently recommended as an additional tool for prognostication because of a wide range of NSE cut-off values^7^. The reasons for the wide range of NSE cut-off values for early prognostication include measurements at different timepoints, the high sensitivity of the measurement to blood sample handling and storage conditions (e.g., haemolysis), the type of assay used, differences in the sensitivity for hypoxia, and the presence of extraneuronal sources of biomarkers^8–12^.

Previous studies have reported that NSE is released from damaged neurons into the cerebrospinal fluid (CSF) and then released into the systemic circulation due to blood–brain barrier (BBB) disruption, and that the CSF NSE prognostic performance was significantly higher than that of serum NSE at 24 h after ROSC and had excellent area under the receiver operating characteristic curve (AUROC) values and a high sensitivity at 100% specificity, although the process of obtaining CSF NSE is invasive^13–18^.

To the best of our knowledge, no studies have evaluated the prognostic value and level of serum NSE in OHCA patients treated with TTM in relation to severe BBB disruption. We evaluated the heterogeneity of serum NSE levels and its prognostic value in predicting poor neurologic outcomes between post-OHCA patients treated with TTM with or without severe BBB disruption.

## Methods

This study was approved by the Institutional Review Board of the Chungnam National University Medical Centre (CNUH IRB 2018-04-051). All procedures and protocols were implemented in accordance with the Declaration of Helsinki and the International Conference of Harmonization and Good Clinical Practice (ICH GCP); they were also reported following the CONSORT criteria. Approval and written informed consent were obtained from the patients’ next-of-kin.

### Study design and patients

This was a prospective, single-centre, observational cohort study of patients who were treated with TTM following OHCA from January 2019 to June 2021. For the primary endpoint, we compared the prognostic value and serum NSE levels between post-OHCA patients treated with TTM with and without severe BBB disruption.

Patients’ neurologic statuses were obtained by directly calling the patient’s caregiver 6 months after ROSC. A cerebral performance category (CPC) of 1–2 demonstrated good neurologic outcomes, while a CPC of 3–5 was related to poor neurologic outcomes. Resuscitated OHCA patients who underwent TTM and whose Glasgow Coma Scale (GCS) score was ≤ 8 following ROSC were included in the study. The exclusion criteria for this study were as follows: (1) < 18 years of age; (2) experienced a traumatic CA or an interrupted TTM (due to haemodynamic instability), (3) ineligibility for TTM (i.e., intracranial haemorrhage, active bleeding, known terminal illness, or poor pre-arrest neurological status); (4) ineligibility for lumbar puncture (LP) (i.e., brain computed tomography showed severe cerebral oedema, obliteration of the basal cisterns, occult intracranial mass lesion, antiplatelet therapy, anticoagulation therapy, or coagulopathy: platelet count < 40 × 10^3^/mL or international normalised ratio > 1.5)^19^, (5) receiving extracorporeal membrane oxygenation, (6) no next-of-kin to consent to LP, and (7) refusal of further treatment by the next-of-kin.

### TTM protocol

TTM was applied using cooling devices (Arctic Sun^®^ Energy Transfer Pads™, Medivance Corp., Louisville, KY). The target temperature of 33 °C was maintained for 24 h with subsequent rewarming to 37 °C at a rate of 0.25 °C/h, and the temperature was monitored using an oesophageal and bladder temperature probe. An Anesthetic Depth Monitor for Sedation (Unimedics Co., Ltd., Seoul, Korea) was used to monitor the anaesthesia depth. Midazolam (0.05 mg/kg intravenous bolus, followed by a titrated intravenous continuous infusion at a dose between 0.05 and 0.2 mg/kg/h) and cisatracurium (0.15 mg/kg intravenous bolus, followed by an infusion of up to 0.3 mg/kg/h) were administered for sedation and control of shivering. Electroencephalography was performed for patients with persistent deterioration of their level of consciousness, involuntary movements, or seizures. If there was evidence of electrographic seizure or a clinical diagnosis of seizure, anti-epileptic drugs were administered (levetiracetam: loading dose, 2 g bolus intravenously; maintenance dose, 1 g bolus twice daily, intravenously). Fluid resuscitation or vasopressors were administered when necessary to maintain a mean arterial pressure between 85 and 100 mmHg^20^.

### Data collection

As in a previous study^17^, the following data were collected from the database: age, sex, presence of a witness at the time of collapse, bystander CPR, first monitored rhythm, aetiology of CA, time from collapse to CPR (no flow time), time from CPR to ROSC (low flow time), time from ROSC to achieving the target temperature of 33 °C (induction time), time from ROSC to obtaining intracranial pressure (ICP) via LP (ICP time), time from ROSC to obtaining blood and CSF via arterial and lumbar catheter (sample time), sequential organ failure assessment (SOFA), GCS scores after ROSC, and CPC at 6 months after ROSC.

### Measurement of albumin quotient and serum NSE

The procedure was performed with the patient lying in the lateral decubitus position. A lumbar catheter was inserted using a Hermetic™ lumbar accessory kit (Integra Neurosciences, Plainsboro, NJ) at the level of the lumbar spine between L3 and L4 in patients whose hips and knees were flexed during the procedure. Blood and CSF were obtained via a radial arterial and lumbar catheter, respectively, on the third day of hospitalisation. Blood collected from the enrolled patients was centrifuged for 10 min at 3000 rpm. The collected serum and CSF were immediately frozen and stored at − 40 °C until analysis by Green Cross Laboratories (GC Labs) (Yongin, Korea). Serum NSE levels were determined using an electrochemiluminescence immunoassay kit (COBAS^®^ e801, Roche Diagnostics, Rotkreuz, Switzerland). Aliquots with haemolysis exceeding a defined threshold value were discarded automatically. The measurement range was 0.1–300 μg/L (normal values: < 16.3 μg/L). At GC Labs, the between-run precision at concentrations of 12.39 and 96.16 μg/L was 1.74% and 1.66%, respectively^18^. Severe BBB disruption was evaluated using CSF serum albumin quotient (Qa) values; CSF serum albumin is the gold standard for the functional assessment of BBB disruption on Day 3 of hospitalisation^16^. Qa values > 0.02 indicated severe BBB disruption^21^.

### Sample size

In a previous study^22^, the range of AUROCs predicting poor neurologic outcomes using serum NSE was 0.77–0.97 in CA patients with TTM, and 11–57 patients were required to achieve a power level of 0.90 at a significance level of 0.05 (two-sided test).

### Statistical analysis

Continuous variables were reported as medians with interquartile ranges or means and standard deviations, depending on normal distribution. Categorical variables were reported as frequencies and percentages. Comparisons between the two groups were made using the chi-squared test, Fisher’s exact test, the Mann–Whitney U test, or the two-tailed t-test. The AUROC was used to identify cut-off values of serum NSE in patients with and without severe BBB disruption for predicting neurologic outcomes. The correlation between Qa, serum, and CSF NSE was analysed using Kendall tau. All statistical analyses were performed using PASW/SPSS software, version 18 (IBM, Armonk, NY) and MedCalc 15.2.2 (MedCalc software, Mariakerke, Belgium). Results were considered statistically significant at P < 0.05 (two-tailed).

## Results

### Characteristics of study subjects

**Figure 1:**
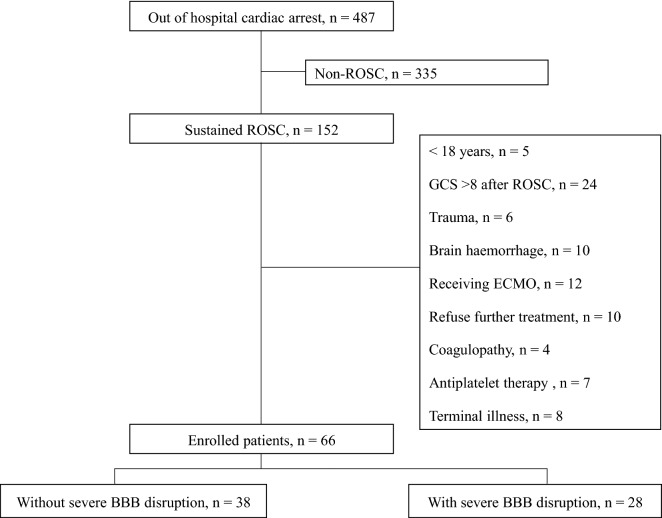
Flowchart of the study (ROSC: return of spontaneous circulation; GCS: Glasgow Coma Scale; ECMO: extracorporeal membrane oxygenation; BBB: blood–brain barrier).

Of 152 post-OHCA patients in whom ROSC was recorded, 66 patients were enrolled in the study (Fig. 1). Serum and CSF samples were obtained 53–57 h after ROSC on day 3 after ROSC. Twelve patients underwent delayed percutaneous coronary intervention to evaluate acute myocardial infarction as the cause of OHCA after TTM in this study. No complications related to the lumbar drainage catheter, including bleeding, infection, or brain herniation, occurred in the enrolled patients. There were no significant differences between patients with and without severe BBB disruption in terms of mean age, sex, witness, bystander CPR, causes of collapse, GCS, induction time, no flow time, ICP time, sample time, or SOFA scores (Table 1). Of the 66 enrolled patients, 27 (40.9%), 4 (6.1%), 0 (0.0%), 22 (33.3%), and 13 (19.7%) had a CPC of 1, 2, 3, 4, and 5, respectively. Fourteen patients (21.2%) had a CPC of 5 with conservative management after TTM was completed in this study. Of these, one patient died through the withdrawal of life-sustaining therapies, eight patients died after organ donation, and five patients died of pneumonia.

**Table 1:** General characteristics.

Characteristics		Total (n = 66)	Without severe BBB disruption (n = 38)	With severe BBB disruption (n = 28)	P value
Age (years)		54.05 ± 17.16	50.82 ± 17.88	58.43 ± 15.37	0.08
Sex, n (%)	Male	50 (75.8)	32 (84.2)	18 (64.3)	0.06
	Female	16 (24.2)	6 (15.8)	10 (35.7)	
Witness, n (%)	Yes	44 (66.7)	23 (60.5)	21 (75.0)	0.22
	No	22 (33.3)	15 (39.5)	7 (25.0)	
Bystander CPR, n (%)	Yes	46 (69.7)	30 (78.9)	16 (57.1)	0.06
	No	20 (30.3)	8 (21.1)	12 (42.9)	
Initial rhythm, n (%)	Asystole	24 (36.4)	10 (26.3)	14 (50.0)	0.04
	PEA	23 (34.8)	12 (31.6)	11 (39.3)	
	VF	17 (25.8)	14 (36.8)	3 (10.7)	
	Pulseless VT	0 (0.0)	0 (0.0)	0 (0.0)	
	Unknown	2 (3.0)	2 (5.3)	0 (0.0)	
Causes, n (%)	Hypoxia	39 (59.1)	22 (59.5)	17 (60.7)	0.91
	MI	12 (18.2)	6 (16.2)	6 (21.4)	
	Arrhythmia	11 (16.7)	7 (18.9)	4 (14.3)	
	Unknown	4 (6.0)	3 (7.9)	1 (3.6)	
GCS, n (%)	3	52 (78.8)	25 (65.8)	27 (96.4)	0.07
	4	4 (6.1)	4 (10.5)	0 (0.0)	
	5	3 (4.5)	3 (7.9)	0 (0.0)	
	6	3 (4.5)	2 (5.3)	1 (3.6)	
	7	2 (3.0)	2 (5.3)	0 (0.0)	
	8	2 (3.0)	2 (5.3)	0 (0.0)	
Neurologic outcome	Good	31 (47.0)	25 (65.8)	6 (21.4)	< 0.001
	Poor	35 (53.0)	13 (34.2)	22 (78.6)	
Induction time (h)		6.37 ± 2.91	6.20 ± 2.63	6.59 ± 3.27	0.60
No flow time (min)		2.00 (0.00, 11.50)	1.00 (0.00, 16.00)	3.50 (0.25, 10.75)	0.38
Low flow time (min)		18.00 (8.50, 28.50)	15.00 (7.00, 23.00)	25.50 (10.25, 39.00)	0.01
ICP time (h)		4.50 (3.22, 6.50)	4.43 (3.17, 6.00)	4.59 (3.23, 6.05)	0.86
Sample time (h)		55.00 (53.00, 57.00)	55.00 (53.00, 57.00)	54.00 (52.25, 56.00)	0.13
SOFA score		12.00 (10.00, 13.00)	11.00 (9.75, 13.00)	12.00 (10.25, 12.75)	0.59
Albumin quotient		0.015 (0.007, 0.031)	0.007 (0.006, 0.012)	0.054 (0.026, 0.156)	< 0.001

Continuous variables are expressed as mean ± standard deviation or median (interquartile range) depending on the normal distribution.

BBB, blood brain barrier; CPR, cardiopulmonary resuscitation; MI, myocardial infarction; GCS, Glasgow Coma Scale; ICP, intracranial pressure; SOFA, sequential organ failure assessment; PEA, pulseless electrical activity; VF, ventricular fibrillation, VT, ventricular tachycardia.

### Comparison of serum NSE levels between groups with and without severe BBB disruption

In patients with good neurologic outcomes, there were no significant differences in serum NSE levels between patients with and without severe BBB disruption. In patients with poor neurologic outcomes, serum NSE levels were higher in those with severe BBB disruption than in those without, although there were no significant differences in CSF NSE levels between groups (Table 2).

**Table 2:** Comparison of serum neuron-specific enolase between groups with and without severe blood–brain barrier disruption.

	Total	Non-severe BBB disruption	Severe BBB disruption	P value
**CSF NSE (μg/L)**				
Good outcome	22.30 (11.30, 59.90)	22.30 (11.15, 53.10)	20.40 (10.44, 154.25)	0.94
Poor outcome	300.00 (300.00, 300.00)	300.00 (300.00, 300.00)	300.00 (298.23, 300.00)	0.83
**Serum NSE (μg/L)**				
Good outcome	22.40 (15.70, 25.00)	22.40 (15.65, 24.95)	22.55 (17.88, 34.53)	0.58
Poor outcome	91.95 (24.55, 216.00)	26.35 (20.30, 84.13)	127.00 (54.65, 300.00)	0.006

Continuous variables are expressed as median (interquartile range) depending on the normal distribution. NSE, neuron-specific enolase; BBB, blood–brain barrier; CSF, cerebrospinal fluid.

### Prognostic value of serum NSE in predicting poor neurologic outcomes in patients with and without severe BBB disruption

**Figure 2:**
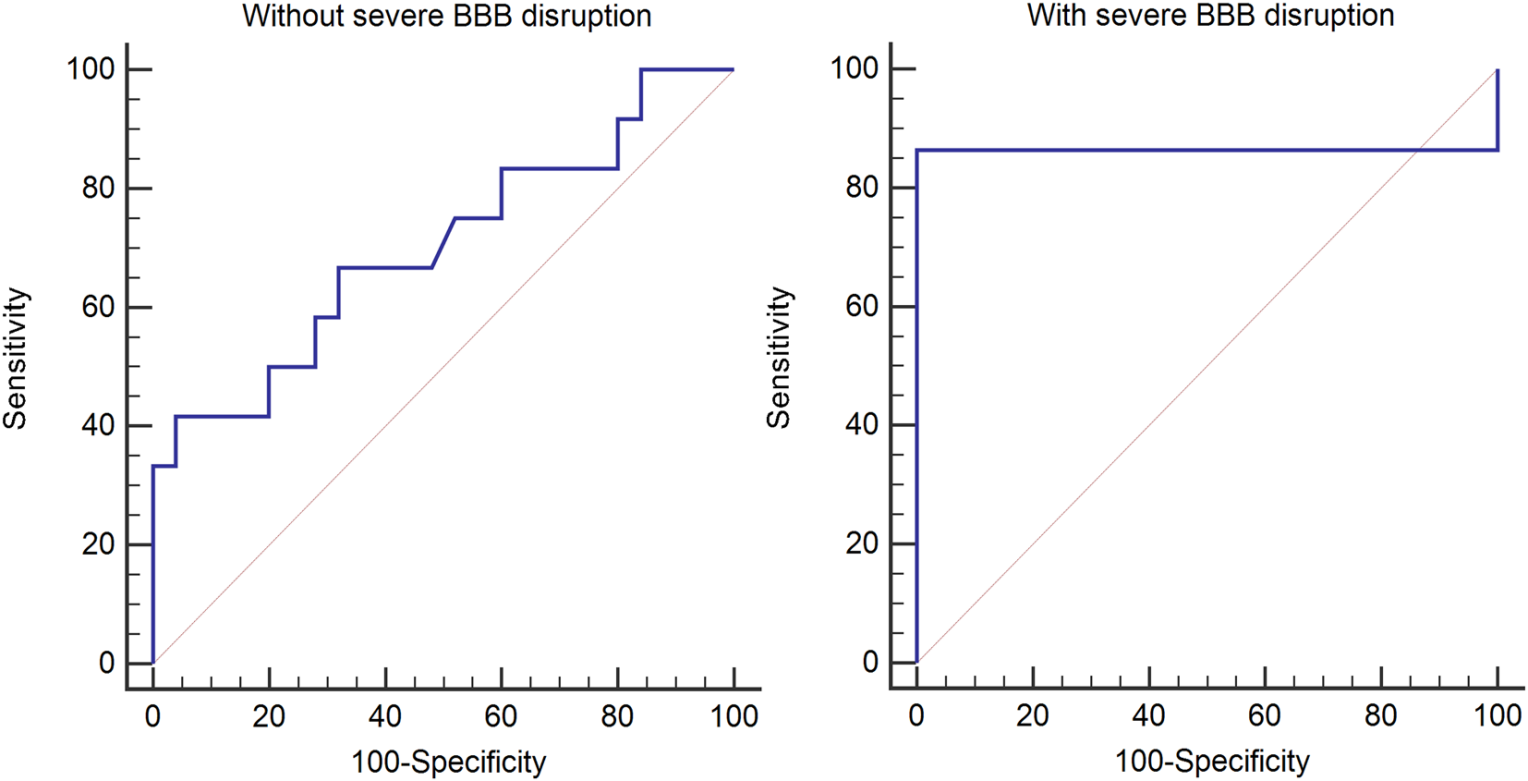
Receiver operating characteristic curves for prediction of poor neurologic outcomes using serum NSE. In the group with severe BBB disruption, the area under the receiver operating characteristic curve for serum NSE showed a better performance than that in the group without severe BBB disruption. (NSE: neuron-specific enolase; BBB: blood brain barrier).

The AUROC of serum NSE in the group with severe BBB disruption was 0.86, whereas that of serum NSE in the group without severe BBB disruption was 0.70. A serum NSE cut-off value of 40.4 μg/L in the group without severe BBB disruption had a sensitivity of 41.7% and a specificity of 96.0%, whereas a cut-off value of 34.6 μg/L in the group with severe BBB disruption had a sensitivity of 86.4% and a specificity of 100.0% (Fig. 2).

### Correlation of serum NSE, CSF NSE, and Qa in patients with and without severe BBB disruption

**Figure 3:**
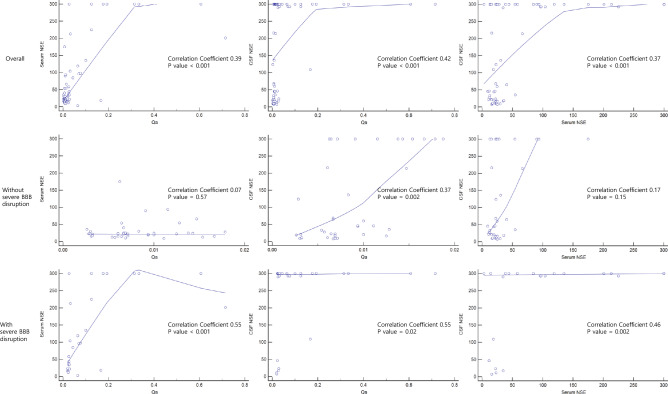
Correlations between Qa, serum NSE, and CSF NSE. In the group without severe BBB disruption, there were no statistically significant correlations between serum and CSF NSE. Meanwhile, in the group with severe BBB disruption, there were large positive correlations between serum and CSF NSE. (NSE: neuron-specific enolase; Qa: CSF serum albumin quotient; CSF: cerebrospinal fluid; BBB: blood brain barrier).

Overall, there were medium or large positive correlations between Qa and serum NSE, Qa and CSF NSE, serum and CSF NSE. In the group without severe BBB disruption, there were no statistically significant correlations between serum and CSF NSE, and Qa and serum NSE; however, the correlation between Qa and CSF NSE was significant. Meanwhile, in the group with severe BBB disruption, there were large positive correlations between Qa and serum NSE, Qa and CSF NSE, serum and CSF NSE (Fig. 3).

## Discussion

The major result of this study was the observation that the association of serum NSE with neurologic outcomes differed significantly, depending on severe BBB disruption. In patients with poor neurologic outcomes, serum NSE levels in the group with severe BBB disruption were higher than those in the group without severe BBB disruption, regardless of CSF NSE levels. In patients with good neurologic outcomes, there was no difference in serum NSE levels between groups with and without severe BBB disruption, as CSF NSE levels were low. Additionally, the prognostic value of serum NSE in the group with severe BBB disruption was more useful than that in the group without severe BBB disruption. Therefore, it is necessary to consider the heterogeneity of groups with and without severe BBB disruption when using a cut-off value of serum NSE as a prognostic predictor, although serum NSE would be useful, regardless of the occurrence of severe BBB disruption.

NSE is a cytoplasmic glycolytic enzyme with a serum half-life of approximately 24–72 h. The enzyme exists as a dimer and has three subunits: α, β, and γ. NSE in the brain has two α and γ subunits, but no β subunits. The dimeric αα form is specific for glial cells, whereas γ-enolase is found in neurons and other cells of neuroectodermal origin. Both the γγ and αγ forms are also present in erythrocytes and platelets. Thus, haemolysis can increase serum levels of NSE in proportion to the degree of haemolysis, even in the absence of brain injury^23^. NSE levels > 33 μg/L measured within 48 h in OHCA patients not treated with TTM had been used as a reliable marker for predicting poor neurologic outcomes^24^. High serum values of NSE at 48–72 h after CA support the prognosis of poor neurologic outcomes, especially if repeated sampling results in consistently high values^7,25^. However, some studies on NSE in CA patients treated with TTM have provided conflicting results for cut-off values for the prediction of poor neurologic outcomes, with no false positives ranging from 28 to > 100 μg/L^4,26–32^. In this study, the cut-off values of serum NSE at 53–57 h after ROSC between groups with and without severe BBB disruption was different, although serum NSE showed a good performance for predicting poor neurologic outcomes.

The BBB regulates the brain parenchymal movement of plasma components. In general, the transcellular pathway of the BBB is migration by passive diffusion. However, only neutral lipophilic substances with a molecular weight < 450 Da can travel through this pathway^33–35^. NSE with a molecular weight of 78 kDa is released into the CSF from damaged neurons and then into the systemic circulation when severe BBB disruption occurs, and is predictive of poor neurologic outcomes^13–16,36^. In this study, the group with severe BBB disruption had significantly higher serum NSE levels and poor neurological outcomes compared to the group with severe BBB disruption. Additionally, in the group without severe BBB disruption, there were no statistically significant correlations between serum and CSF NSE. Meanwhile, in the group with severe BBB disruption, there were large positive correlations between serum and CSF NSE, although the actual correlation coefficient would be higher, considering that the maximal measurement range of NSE was 300 μg/L in this study.

There are several limitations to this study. First, this was a single-centre study with a small sample size that might limit the generalisability of our findings, although a total of 57 patients were required for the study. Second, CSF albumin was obtained through a lumbar catheter. One study reported that albumin concentration in the lumbar space was 2.2 times higher than that in the ventricle^37^. However, the heterogeneity of both groups with and without severe BBB disruption was observed as the purpose of this study. Third, as the maximal measurement range of NSE was 300 μg/L in this study, an accurate trend of serum NSE could not be predicted. Fourth, we did not assess the histopathology, cellular findings, or neuroimaging findings in the brain cortex at 24 h after ROSC. However, this study revealed that severe BBB disruption affects the prognostic value of serum NSE. Fifth, NSE was measured on the third day of hospitalisation; therefore, it was impossible to determine the change in NSE over the long term. Sixth, other biomarkers such as neurofilament light chain were not measured; hence, we could not speculate on the changes in these parameters. Seventh, DCE-MRI or S 100b protein were not used for evaluating severe BBB disruption. However, the Qa used in this study is known as the gold standard for the functional assessment of BBB disruption^16,38,39^. Additionally, the purpose of this study was not to reveal BBB disruption, but to reveal that severe BBB disruption is one of the reasons why the cut-off value of NSE, which is known to be useful, has a wide range in predicting the neurological prognosis of OHCA patients treated with TTM. Finally, the investigator was not blinded throughout the experiment. Future studies involving blinding are needed to focus on this limitation.

## Conclusions

In evaluating the cut-off and prognostic value of serum NSE as a predictor of poor neurologic outcomes in OHCA patients treated with TTM, we demonstrated that patients with and without severe BBB disruption were heterogeneous. However, as the method of using the CSF serum albumin quotient to determine the presence or absence of severe BBB disruption is invasive, further studies on how to detect severe BBB disruption are needed to present the cut-off value of serum NSE as a prognostic predictor in OHCA patients treated with TTM.
